# Biomarkers of Aging–NIA Joint Symposium 2024: New Insights Into Aging Biomarkers

**DOI:** 10.1111/acel.70124

**Published:** 2025-06-17

**Authors:** Chiara Herzog, Jesse R. Poganik, Nir Barzilai, Nathan Basisty, Isabel Beerman, Daniel W. Belsky, Rafael de Cabo, Julián Candia, Faraz Faghri, Steve Horvath, Andrea B. Maier, Viviana Perez, Payel Sen, Mahdi Moqri, Vadim N. Gladyshev, Luigi Ferrucci

**Affiliations:** ^1^ Department of Twin Research and Genetic Epidemiology King's College London London UK; ^2^ European Translational Oncology Prevention and Screening Institute Universität Innsbruck Innsbruck Austria; ^3^ Division of Genetics, Department of Medicine Brigham and Women's Hospital, Harvard Medical School Boston Massachusetts USA; ^4^ American Federation for Aging Research (AFAR) Albert Einstein College of Medicine New York USA; ^5^ Intramural Research Program National Institute on Aging, National Institutes of Health Baltimore Maryland USA; ^6^ Robert N. Butler Columbia Aging Center Columbia University Mailman School of Public Health New York New York USA; ^7^ Center for Alzheimer's and Related Dementias National Institute on Aging, National Institutes of Health Bethesda Maryland USA; ^8^ DataTecnica Washington, D.C USA; ^9^ Laboratory of Neurogenetics National Institute on Aging, National Institutes of Health Bethesda Maryland USA; ^10^ University of California Los Angeles California USA; ^11^ Altos Labs Cambridge UK; ^12^ Department of Human Movement Sciences, @AgeAmsterdam, Faculty of Behavioural and Movement Sciences Vrije Universiteit Amsterdam, Amsterdam Movement Sciences Amsterdam the Netherlands; ^13^ NUS Academy for Healthy Longevity, Yong Loo Lin School of Medicine National University Singapore Singapore; ^14^ Hevolution Foundation Riyadh Saudi Arabia; ^15^ Laboratory of Genetics and Genomics National Institute on Aging, National Institutes of Health Baltimore USA

## Abstract

The second Biomarkers of Aging Symposium, jointly hosted by the National Institute on Aging (NIA) Intramural Research Program and the Biomarkers of Aging Consortium (BAC) on September 12, 2024, in Baltimore, MD, convened leading researchers, clinicians, and stakeholders in the aging field to share new developments and discuss roadmaps to advance biomarkers of aging. This meeting report summarizes the highlights of this symposium and underscores the urgent need to understand longitudinal, complex, and heterogeneous processes of aging to unlock the full potential of aging biomarkers.

## Graphical abstract

Summary of key points raised by the Biomarkers of Aging–NIA Symposium 2024.

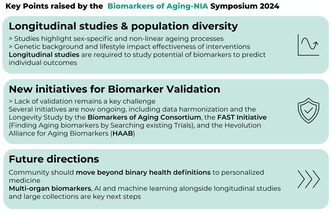

Biomarkers of aging are quantifiable measures that predict biological age and ideally its changes in response to interventions (Moqri et al. 2023). Such measures are critically needed for studies on human aging and responses to geroprotective interventions owing to their potential to rapidly indicate an intervention's efficacy prior to long term clinical outcomes (as validated surrogate endpoints), minimize the sample size required to show effectiveness, and predict an individual's future risk of disease. Advances in omics technologies and ever‐growing big data from human and animal studies have catalyzed biomarker development in recent years, and a large number of studies have identified increasingly accurate candidate biomarkers capable of predicting biological age, lifespan, morbidity, and/or age‐related disease(s). Despite this remarkable progress, systematic validation of biomarkers of aging for clinical use has remained elusive, representing a considerable barrier to both basic and translational research (Khan et al. 2024). The symposium explored the current progress and open challenges from diverse angles, ranging from basic research to clinical practice and public perception.

## 1 Investigating Underlying Processes and Conserved Mechanisms to Derive Informative Biomarkers of Aging

Dan Belsky (Columbia University) highlighted current challenges and future directions in algorithm‐based aging biomarker development with a focus on the study designs used to derive these biomarkers. As reviewed by the Biomarkers of Aging Consortium, the current state‐of‐the‐art biomarkers of aging are algorithms derived from machine‐learning (ML) analyses of “omics” datasets (e.g., DNA methylation, proteomics, metabolomics, and others) (Moqri et al. 2023; Rutledge et al. 2022). These biomarkers are generated by fitting predictive models to proxy measurements of biological aging. Because there is no gold‐standard measurement of biological aging (Ferrucci et al. 2020) nor consensus about what one should be (Gladyshev et al. 2024), these proxy measurements vary from study to study. While the most common proxy is calendar age (time since birth), some algorithms are trained to predict survival (time until death; e.g., GrimAge (Lu et al. 2019)), healthspan (time until incidence of first chronic health condition; e.g., PhenoAge (Levine et al. 2018)), or pace of aging (rate of decline in multiorgan system integrity; e.g., DunedinPACE (Belsky et al. 2022)). Many of the resulting algorithmic biomarkers, especially those trained to predict survival, healthspan, or pace of aging, meet a range of validation criteria proposed for biomarkers of aging (Moqri et al. 2024a). However, the relationship between the molecular elements that form these algorithms and the underlying biological processes of aging they are designed to represent remains unclear. They may represent causes of aging‐related decline in the integrity and resilience capacity of cells, tissues, and organs. Or, alternatively, they may reflect the consequences of such decline. Current approaches are likely to produce algorithmic biomarkers comprising a mixture of these types of molecular elements. To delineate the ways in which individual molecular measurements relate to processes of biological aging, data integration is needed across multiple research designs: predictive modeling of biological aging proxies (the current approach), in vitro experiments to establish mechanistic relationships of molecular measurements with aging processes (the approach described by Ferrucci, see below), and responsive modeling of effects of interventions known to manipulate processes of biological aging (the approach outlined by Barzilai for AFAR's “FAST” initiative, see section on multiomics below). The next steps for aging biomarker development are to accelerate in vitro experimental and response‐biomarker analyses to enable such evidence triangulation.

Luigi Ferrucci (NIA) emphasized the potential of linking molecular aging biology to biomarkers, which could help to develop more informative biomarkers, with the example of mitochondrial dysfunction: both published and unpublished data from the Baltimore Longitudinal Study of Aging (BLSA) suggest that decreasing mitochondrial capacity with age is involved in human age‐related muscle and cognitive decline. Importantly, metabolomic markers of mitochondrial function, such as glycerophospholipids that play a role in maintaining mitochondrial integrity, can predict cognitive and mobility decline, alongside several other key age‐related diseases (Tian et al. 2024). This research represents a systematic approach for connecting biomarkers to underlying biology and vice versa. In parallel, Rafael de Cabo (NIA) outlined how studies in animal models such as the comprehensive Study of Longitudinal Aging in Mice (SLAM) (Palliyaguru et al. 2021), initiated in 2015, can provide rich datasets to investigate underlying aging biology in parallel to human studies such as BLSA. While conducted in a single‐site manner, in contrast to ITP, and although animal models in highly controlled environments may not always reflect all aging processes in humans, they provide valuable insights into conserved aging mechanisms shared across mammals that can be later validated in human studies including comparable measures. The importance of comparing aging biology and biomarkers across species was also echoed by Steve Horvath (Altos Labs), who introduced a pan‐mammalian DNA methylation‐based epigenetic clock (Lu et al. 2023) as well as recent clocks comparing epigenetics in the highly regenerative and long‐lived axolotl and humans (Haluza et al. 2024). Steve Horvath also presented other recent advances in epigenetic clock research, focusing on longitudinal studies, rejuvenation interventions, conceptual frameworks, and newly developed epigenetic clocks. For instance, a recent preprint from the InCHIANTI cohort demonstrated that longitudinal changes in epigenetic clocks predict survival outcomes (Kuo et al. 2024), providing evidence that so‐called “second generation” clocks ‐ which track outcomes other than chronological age ‐ may have the potential to capture causal aspects of aging. Intriguingly, emerging literature suggests some interventions could rejuvenate the epigenetic clock—including metformin and IL‐11 signaling inhibitors (Widjaja et al. 2024)—but despite these advances, Horvath pointed out that the field suffers from inconsistent use of epigenetic clocks. To address this issue, he proposed three strategic approaches for clock selection: 1) evaluate multiple clocks simultaneously and report all results; 2) select a clock based on community consensus and benchmarking studies; and 3) choose a clock based on its biological relevance to the mechanism of interest. Examples supporting the third approach include the PRC2‐AgeIndex (Moqri et al. 2024b), proposed as a universal biomarker of aging and rejuvenation; a multispecies clock designed to enhance translational relevance (Lu et al. 2023); or causality‐enriched epigenetic age models, which uncouple damage and adaptation (Ying et al. 2024a).

Horvath also discussed factors that may influence epigenetic age, including proliferative activity and turnover rates (Gorelov et al. 2024), which could explain why some tissues—such as the human breast—may exhibit older epigenetic ages than other tissues (Horvath 2013). Recent models have also explored conceptual and mathematical models underlying epigenetic clocks. Horvath referenced a 2024 study by Tong et al. (Tong et al. 2024), which showed that up to 75% of the accuracy of Horvath's pan‐tissue clock could be explained by stochastic processes, whereas the remaining 25% reflects deterministic signals, providing insights into nonrandom biological aging mechanisms. Horvath concluded that the most recent clocks, including those reflecting histone marks (de Lima Camillo et al. 2025) or based on large language models (LLMs) (de Lima Camillo et al. 2024; Ying et al. 2024b) could present exciting advancements in the field, potentially paving the way for more accurate biological age estimation.

Further insights into epigenetic alterations and their link to other molecular alterations with aging, including a loss of gene expression control of developmental and inflammatory genes, were shared by Payel Sen (NIA). Sen emphasized the importance of integrating outputs from multiple “omic” layers and tracing these over time. Some early molecular changes such as chromatin accessibility may not immediately reflect on gene expression. Others, like DNA methylation, may be established late. Single‐cell multiomic profiling and H3K27me3 patterns in aged tissues suggest that cellular identity becomes less distinct with aging. These insights offer a mechanistic basis for understanding how molecular alterations may drive age‐related phenotypes.

## 2 The Need for Longitudinal Studies in Aging Research

Much of the research into the underlying biology of aging presented at the meeting was uniquely facilitated by longitudinal insights, derived from studies such as BLSA and SLAM. Accordingly, a recurring issue raised by nearly every presenter was the growing need for longitudinal studies in aging research to accurately reflect dynamic individual trajectories. For instance, Toshiko Tanaka and Rafael de Cabo (both NIA) argued that longitudinal data allow for stronger associations of changes with physical and cognitive health in comparison to cross‐sectional phenotypes (Kuo et al. 2022), emphasizing the value of longitudinal investigations.

## 3 Untangling Heterogeneity: Genetics, Sex, and Population

Genetic background, sex, and population factors such as race and ethnicity can influence aging trajectories, evidenced by both human and animal studies—notably in particular with the help of longitudinal data: findings from SLAM have shown sex‐ and strain‐specific aging patterns, underscoring the limitations of cross‐sectional studies, which often fail to capture such features (de Cabo, NIA). As blood is an easily accessible resource, making it attractive for potential biomarkers, it is important to establish the potential differences between these variables (including genetic diversity and sex). Isabel Beerman (NIA) explained that while many blood cell parameters have concordant changes associated with aging between sexes in mice, the degree of change within different blood cell parameters is divergent between males and females. Furthermore, the rates of change are often nonlinear—requiring comprehensive analysis of multiple age groups to better classify distinct, sex‐specific inflection points. These concerns were further validated by Nir Barzilai (Albert Einstein College of Medicine), who also reported nonlinear alterations in the plasma proteome with age that were dependent on genetic heritage (i.e., being an offspring of parents with exceptional longevity) and sex. Together, these findings suggest that customized clocks for different population groups may need to be developed. This does not preclude the potential for a universal set of aging biomarkers established from easily accessible tissue and relatively inexpensive assays, including complete blood counts, but we need to ensure that the parameters included fully capture the spectrum of diverse aging phenotypes.

Diversity with relation to aging biomarkers and longevity extends beyond biology, as Andrea Maier (NUS) pointed out. The HEalthy LOngevity (HELO) Consortium (https://helo‐nus.com/), led by the National University of Singapore, is surveying the general population in multiple countries, including Singapore, Indonesia, the Netherlands, Switzerland, Hungary, the United States, the United Arab Emirates, and Thailand, to better understand the relevance and consequences of current and predicted future changes in lifespan, healthspan, and healthy longevity for public health. Nearly 70% of Singaporeans surveyed expressed an interest in visiting healthy longevity medicine clinics, compared to only 10% in neighboring Indonesia, pointing to significant regional differences in cultural sensitivity toward themes related to longevity.

## 4 Choosing the Right System to Measure: Does One Size Fit All?

In addition to diversity across individuals, it is becoming increasingly evident that aging within individuals may likewise not occur at uniform rates, as highlighted by Vadim Gladyshev (Harvard University). Factors may affect different organs to different extents, highlighting the immense complexity of the aging process. Biomarkers that offer insights into multiple organ systems from a single blood sample, such as recently described proteomic organ‐specific clocks, may help to tease apart effects of exposures and interventions on different organs (Goeminne et al. 2024; Oh et al. 2023).

In his efforts to identify proteomics‐based biomarkers of senescent cell burden, Nathan Basisty (NIA) discussed the benefits and challenges of using blood as a source for aging biomarkers: While routinely collected for biobanks and trials—and often available longitudinally—blood samples as a source of biomarkers also come with certain challenges. These include a lack of spatial resolution, complex matrix effects due to the coexistence of a large dynamic range of proteins and metabolites, and a lack of tissue specificity—although the latter may be overcome by devising organ‐specific clocks. Monitoring aging across multiple tissues may also improve the understanding of aging biology: for instance, a combination of bone marrow and blood aging can improve age prediction in mice (Beerman, NIA). Nonetheless, such research is ultimately conducted with the vision of leveraging blood analysis to inform biology within the bone marrow compartment, given the limitations of obtaining bone marrow as a routine biospecimen.

## 5 Multiomics Data Harmonization in the Spotlight of New Initiatives

The meeting featured presentations of several new initiatives designed to address challenges and advance aging biomarkers.

The Biomarkers of Aging Consortium, represented by Mahdi Moqri, Jesse Poganik, and Vadim Gladyshev (all Harvard University), was initiated in 2022 to establish reliable biomarkers of aging for longevity interventions and has since then implemented several programs to serve the community of aging researchers and biomarker developers. Jesse Poganik shared results from a recent questionnaire that resulted in consensus expert recommendations (Herzog et al. 2024) to accelerate biomarker translation. Recommendations included—amongst others—improving data sharing and fostering collaboration across diverse stakeholders, including biomarker developers and clinicians, for instance to link biomarkers to functional outcomes. Harmonization across multiple datasets and cohorts is a pivotal step toward a better understanding of biomarkers and their validation. Mahdi Moqri shed light on additional Consortium programs to facilitate biomarker harmonization—yet another recurring focal point of discussion throughout the meeting—including Biolearn (Ying et al. 2023). Biolearn is an open‐source library unifying public datasets, analysis methods, and the largest collection of aging biomarkers to date. These harmonized biomarkers and data allow for cross‐population benchmarking and technical validation of AI/ML‐based biomarkers. In addition to the unmet need for harmonization, validation of biomarkers is currently hampered by access to open‐source datasets. To start addressing this problem, the Biomarkers of Aging Consortium is therefore initiating a new longitudinal longevity study (https://longevity.bwh.harvard.edu/), comprising deep multiomic profiling alongside phenotypic and functional data collection. In addition to providing longitudinal profiling data openly, the study team also makes available protocols and standardized questionnaires to facilitate decentralized collections across the world.

Finding Aging biomarkers by Searching existing Trials (FAST) Initiative was created as introduced by Nir Barzilai and Dan Belsky. FAST aims to discover healthspan extension biomarkers by conducting omics assays and analysis of biospecimens collected in completed randomized controlled trials (RCTs) of drugs already approved by the US Food and Drug Administration (FDA). FAST's approach is motivated by three premises: 1) FDA‐approved drugs show evidence of modifying aging biology and extending human healthspan. Barzilai and colleagues identified four FDA‐approved drugs that (a) modify aging biology and increase healthspan and lifespan in laboratory models; and (b) extend healthspan and lifespan in humans: Metformin, SGLT‐2 inhibitors, GLP‐1 agonists, and Bisphosphonates (Kulkarni et al. 2022; Leone and Barzilai 2024); 2) Completed trials of these drugs include outcomes already recognized by FDA, meaning that biomarkers identified as mediating treatment effects on these outcomes will already be candidates for FDA surrogate endpoint status; 3) Omics data reflect aging biology: By integrating results across trials of multiple drugs with different mechanisms of action, Barzilai proposed we can triangulate novel, generalizable biomarkers of healthspan extension.

Finally, Viviana Perez (Hevolution) introduced the Hevolution Alliance for Aging Biomarkers (HAAB). Across two phases, this program sets out to generate extensive multiomic data from selected longitudinal cohort studies meeting specific criteria (including serial measurements, clinical and functional outcomes, with an emphasis on diverse population and several sample types) and establish infrastructure to create a harmonized data superset, open to sharing within 1 year of its construction. In establishing this initiative with specific cohort criteria, HAAB intends to tackle many of the above‐mentioned challenges in aging research—linking biomarkers to function, longitudinal datasets, and diversity—and is mirroring existing successful open datasets such as those built by the Global Neurodegeneration Proteomics Consortium (GNPC).

## 6 Increasing Use of Artificial Intelligence

In line with the generation of new multiomic, multimodal, and longitudinal datasets, such as the Longevity Study and HAAB, the demands for analysis tools are growing, too. Several presentations at the symposium reflected on the increased use of artificial intelligence, systems approaches, or newer and more complex foundation models—like those underlying powerful tools such as ChatGPT. Faraz Faghri (NIA) discussed the role of artificial intelligence and data science approaches in the context of the Centre for Alzheimer's and Related Dementias (CARD) Initiative for both data harmonization and creation of aging models and knowledge engines. Intriguingly, while omics data have been comparatively easy to harmonize in his experience, Faghri noted challenges in harmonizing clinical data for interoperability. Within CARD, the team uses AI to construct models to study the aging brain by integrating multimodal data including imaging. One step further, the use of foundation models was also discussed to build large language models to assist in biomedicine. Julián Candia (NIA) introduced the use of complex system computational approaches toward understanding multifaceted aging biology. While traditional approaches to measure biological aging focus on specific molecules or omics‐based signatures, novel molecular biomarkers of aging may be derived from leveraging interdisciplinary approaches based on complex systems theory and computational tools (Cohen et al. 2022). He discussed two examples, namely: 1) comparing structural properties of transcript and protein co‐regulation networks (Ferrucci et al. 2023), and 2) assessing longitudinal stoichiometry loss in protein complexes (Janssens et al. 2015). Julián Candia concluded by noting that, by analizing structural properties of complex networks, it is possible to infer functional characteristics. In this vein, a promising new line of research is to describe resilience and frailty as emergent, collective properties of the networked system, which switch from one to the other as a phase transition.

The presentations were followed by a roundtable discussion where the need for funding further research in biomarkers of aging was highlighted by invited attendees, including Andrew Brack (ARPA‐H), Felipe Sierra (Hevolution), and Dane Gobel (Methuselah)

## 7 Summary

The second Biomarkers of Aging Symposium provided a platform for important discussions on the status quo and future directions of research to advance biomarkers (see Graphical Abstract). This meeting underscored the need for collection of longitudinal data from diverse populations and reflected on the heterogeneity of aging processes both across and within individuals. A special focus was placed on understanding conserved aging biology leveraging new tools, including multiomics and increased use of artificial intelligence.

Moving ahead, it seems clear that with increasing alignment and collaboration, the field can move forward to tackle large challenges ahead more effectively and establish groundbreaking forward‐looking initiatives, including HAAB, FAST, and the Longevity Study.

Looking ahead toward clinical implementation, Andrea B. Maier finally emphasized the importance of evidence‐based and standardized programs across healthy longevity clinics. Luigi Ferrucci closed with the notion that we need to embrace complexity and move beyond binary health in medicine: currently, an individual is defined as either healthy or sick, yet we know that aging processes are progressive and on a spectrum. It is up to future research to better define “health” in asymptomatic individuals to expand healthspan.

## Author Contributions

**Nir Barzilai:** writing – review and editing. **Rafael de Cabo:** writing – review and editing. **Nathan Basisty:** writing – review and editing. **Mahdi Moqri:** supervision, writing – review and editing. **Viviana Perez:** writing – review and editing. **Faraz Faghri:** writing – review and editing. **Luigi Ferrucci:** supervision, writing – review and editing. **Isabel Beerman:** writing – review and editing. **Chiara Herzog:** writing – original draft, writing – review and editing. **Andrea B. Maier:** writing – review and editing. **Julián Candia:** writing – review and editing. **Daniel W. Belsky:** writing – review and editing. **Payel Sen:** writing – review and editing. **Vadim N. Gladyshev:** supervision, writing – review and editing. **Jesse R. Poganik:** writing – original draft, writing – review and editing. **Steve Horvath:** writing – review and editing.

## Conflicts of Interest

This meeting was supported, in part, by the Intramural Research Program of the National Institute on Aging, NIH, Baltimore, MD. D.W.B. is an inventor of DunedinPACE, which is licensed to TruDiagnostic. Faraz Faghri's participation in the project presented by him was part of a competitive contract awarded to DataTecnica LLC by the National Institutes of Health to support open science research. The Regents of the University of California are the sole owners of patents and patent applications directed at epigenetic biomarkers for which Steve Horvath is a named inventor; S.H. is a founder and paid consultant of the nonprofit Epigenetic Clock Development Foundation that licenses these patents. S.H. is a Principal Investigator at the Altos Labs, Cambridge Institute of Science, a biomedical company that works on rejuvenation.

## Data Availability

Data sharing is not applicable to this article as no new data were created or analyzed in this study.
